# Macroecological scale effects of biodiversity on ecosystem functions under environmental change

**DOI:** 10.1002/ece3.2036

**Published:** 2016-03-16

**Authors:** Hugh M Burley, Karel Mokany, Simon Ferrier, Shawn W Laffan, Kristen J Williams, Tom D Harwood

**Affiliations:** ^1^ Centre for Ecosystem ScienceSchool of Biological, Earth and Environmental SciencesUniversity of New South WalesSydneyNew South Wales2052Australia; ^2^ CSIRO Land and WaterCanberraAustralian Capital Territory2601Australia

**Keywords:** Alpha diversity, beta diversity, complementarity, ecosystem functions, environmental change, functional traits, spatiotemporal analysis

## Abstract

Conserving different spatial and temporal dimensions of biological diversity is considered necessary for maintaining ecosystem functions under predicted global change scenarios. Recent work has shifted the focus from spatially local (*α*‐diversity) to macroecological scales (*β*‐ and *γ*‐diversity), emphasizing links between macroecological biodiversity and ecosystem functions (MB–EF relationships). However, before the outcomes of MB–EF analyses can be useful to real‐world decisions, empirical modeling needs to be developed for natural ecosystems, incorporating a broader range of data inputs, environmental change scenarios, underlying mechanisms, and predictions. We outline the key conceptual and technical challenges currently faced in developing such models and in testing and calibrating the relationships assumed in these models using data from real ecosystems. These challenges are explored in relation to two potential MB–EF mechanisms: “macroecological complementarity” and “spatiotemporal compensation.” Several regions have been sufficiently well studied over space and time to robustly test these mechanisms by combining cutting‐edge spatiotemporal methods with remotely sensed data, including plant community data sets in Australia, Europe, and North America. Assessing empirical MB–EF relationships at broad spatiotemporal scales will be crucial in ensuring these macroecological processes can be adequately considered in the management of biodiversity and ecosystem functions under global change.

## Introduction

The study of *ecosystem functions* or *processes,* defined as stocks and fluxes of matter and energy derived from biological activity (Ghilarov 2000), has received considerable attention in recent decades. While environmental conditions most directly affect these stocks and fluxes, the potential influence of biodiversity on ecosystem functions (B–EF relationships) is also considered important (see Table 1 for definitions of all terms used in this article). Although a substantial body of research has investigated the effects of different dimensions of biodiversity (i.e., taxonomic, functional, and phylogenetic) on the magnitude and stability of key ecosystem functions (Cardinale et al. 2012), the results are often equivocal and controversial (Schwartz et al. 2000). This work has focused primarily on local‐scale manipulative experiments (Gross et al. 2014) or simulations (Loreau and De Mazancourt 2013). The effect of the biological variation at single locations on a range of ecosystem functions is typically assessed over relatively short periods (<10 years) using small numbers of species or biological types. This is essentially an aspatial, “*α*‐diversity” perspective, focusing on the effect of the number of unique biological types within local communities, most commonly floristic species richness at ecological sites. Local‐scale B–EF research has been important in highlighting the potential consequences for ecosystem functions following the expected local biodiversity losses under global change (Cardinale et al. 2012).

**Table 1:** Definitions for key terms used in this article

Term	Definition
Ecosystem functions	Stocks and fluxes of matter and energy derived from biological activity (Ghilarov 2000), for example, primary productivity, evapotranspiration, decomposition (i.e., *ecosystem functions* and *processes* are synonymous). Definitions of “ecosystem services” vary, but generally constitute those “provisioning” or “regulating” ecosystem functions valued by society (e.g., food, water quality; Cardinale et al. (2012)). Here, we focus solely on ecosystem functions.
B–EF (biodiversity–ecosystem function) studies	The study of relationships between different components of biological diversity as explanatory variables (Cardinale et al. 2012) and ecosystem functions as response variables.
*α*‐diversity	The number of biological types – taxonomic, functional, or phylogenetic – found at a particular location (i.e., an ecological plot).
*β*‐diversity	The turnover in biological types (i.e., change in biological composition) between locations over space and or time, both across biogeographic regions, and across entire continents. *β*‐diversity may be quantified as a single measure for a whole region (Whittaker 1960) or as a unique value for every pair of locations (Sørensen 1948).
*γ*‐diversity	The total number of biological types in a region (e.g., all vascular plant species in California), being a function of both *α*‐diversity and *β*‐diversity (Whittaker 1960). *β*‐ and *γ*‐diversity thus constitute the macroecological scale of biodiversity in this article, related to, yet distinct from, diversity at the local scale. *Macroecological diversity* is used synonymously with *β*‐ and *γ*‐diversity in this article.
Functional traits	Any aspect of an organism's phenotype which impacts fitness indirectly via its effects on growth, reproduction, and survival (Violle et al. 2007). Functional traits can influence both the effect of an organism on ecosystem functions (functional effect traits, e.g., organism size) and the organism's response to environmental changes [response traits, e.g., fire response, Mori et al. (2013)].
MB–EF studies	The study of relationships between biodiversity at macroecological scales as explanatory variables (i.e., *β*‐ and *γ*‐diversity across biogeographic regions and continents) and ecosystem functions as response variables.
Macroecological complementarity	The hypothesis that biologically heterogeneous regions with high *β*‐diversity, populated by physiological specialists, display greater magnitudes of ecosystem functions under current conditions than regions where generalists dominate.
Macroecological spatiotemporal compensation	The hypothesis that high *β*‐ and *γ*‐diversity will facilitate spatiotemporal biological exchanges (Loreau et al. 2003) between local communities within a region when environments fluctuate, maintaining regional stability and magnitudes of ecosystem functions under environmental change (Wang and Loreau 2014).

Recent work has extended the B–EF framework by highlighting the potential importance of biodiversity at broader spatial scales in influencing ecosystem functions. Gamma diversity (*γ*), the total number of biological types in a biogeographic region (Table 1), is a function of both *α*‐ and *β*‐diversity (Whittaker 1960). *β*‐diversity can be defined in many ways for different purposes (Baselga 2010; Tuomisto 2010a,b; Barton et al. 2013). Nonetheless, these definitions are unified by the concept of *biological dissimilarity*, which is inherently spatial, being derived from the proximity and connectivity between locations over space and time. The most important aspect of *β*‐diversity for ecosystem functions is the spatial and temporal turnover in biological composition within and between locations across a biogeographic region (Table 1). This is because of the biogeographic processes that structure turnover, which are otherwise difficult to measure across broad spatiotemporal extents, could play important roles in influencing ecosystem functions. Experimental analyses have shown positive effects of *γ*‐ and *β*‐diversity on multiple regional‐level ecosystem functions (Pasari et al. 2013), while simulations have linked greater *β*‐diversity to more stable regional ecosystem functions (Wang and Loreau 2014). However, extending this initial research to achieve a broader understanding of links between “macroecological biodiversity” (*β*‐ and *γ*‐diversity) and ecosystem functions (MB–EF relationships) in natural ecosystems, and to thereby inform real‐world management decisions, will require a new focus from ecologists.

The argument has now been made that conserving biodiversity at all threes scales (*α*,* β*, and *γ*) could have practical, positive implications for landscape management strategies to maintain the stability of future ecosystem processes (Cardinale et al. 2012; Pasari et al. 2013; Wang and Loreau 2014; Isbell et al. 2015). Similarly, it has been previously suggested that rapid species turnover under changing environmental conditions could salvage the contentious prediction that *α*‐diversity maximizes the magnitude and stability of ecosystem functions (Schwartz et al. 2000). Under rapid environmental change, managers will be increasingly required to decide which actions to implement at particular locations across large jurisdictions to achieve different objectives. Local‐scale B–EF research provides some general guidance relevant to managing ecosystem functions within individual areas, such as promoting the maintenance of functional diversity within a site (Cardinale et al. 2012). Extending this research to macroecological scales (Pasari et al. 2013; Wang and Loreau 2014) may provide greater potential for developing modeling approaches to make predictions across entire regions. Such approaches could account for large changes in distributions expected for some species under climate change, along with changes in the composition of communities, and the subsequent effects of these changes on ecosystem functions. Importantly, existing MB–EF studies implicitly assume that these relationships are positive and that they generally hold true in real ecosystems. However, they have only been analyzed in controlled or simulated settings (Pasari et al. 2013; Wang and Loreau 2014).

Before management applications can even be considered, MB–EF research needs further development in several key respects. In this paper, we identify the major challenges in testing and characterizing MB–EF relationships under plausible bioclimatic change scenarios, using data from multiple biological dimensions – taxonomic, functional, and phylogenetic. Incorporating these data sources will provide the foundation for modeling ecosystem functions across broad spatiotemporal extents (Section “Making MB–EF Relationships Applicable to Real Ecosystems”). Development of this capability first requires describing testable hypotheses for current and future MB–EF relationships: “macroecological complementarity” and “macroecological spatiotemporal compensation” (Section “A Broader View of MB–EF Relationships in Natural Ecosystems”). The importance of considering ecological context when assessing these hypotheses in natural systems is illustrated here using a simple practical example of tree communities across an altitudinal transect, where macroecological diversity is hypothesized to drive broad‐scale biomass (Section “An Example: Potential MB–EF Relationships for Trees Across an Elevation Gradient”). Finally, we outline the main avenues, potential methods, and example data sources for testing both MB–EF mechanisms in real ecosystems (Section “Potential Avenues for Testing MB–EF Mechanisms in Real Ecosystems”).

## Making MB–EF Relationships Applicable to Real Ecosystems

Macroecological analyses that consider likely outcomes for ecosystem functions under a plausible range of current and future bioclimatic change scenarios would provide a more robust test of the B–EF concept as a whole. Importantly, initial work on the MB–EF concept has considered only short term or random variation in environmental conditions and has simulated, or controlled for, changes in biological composition (Loreau and De Mazancourt 2013; Pasari et al. 2013; Wang and Loreau 2014). This is despite strong evidence that future bioclimatic shifts are likely to be spatiotemporally directional, auto‐correlated, and at least partly deterministic (Barton et al. 2015; Oliver et al. 2015). For example, observations and predictions for Australia indicate that rainfall will continue to decrease across southwestern biogeographic regions (Suppiah and Hennessy 1998; Gallant et al. 2007, 2013), while increasing in northwestern regions. These shifts will significantly impact the distributions of both individual species and ecological communities [e.g., alterations in competitive regimes between C3 and C4 grasses, (Hughes 2003), and poleward shifts in avian species (Vanderwal et al. 2013)]. Similarly, direct human modifications, such as land clearing, are focused in particular biogeographic regions or landforms. Therefore they have nonrandom impacts on macroecological biodiversity patterns and processes (Cardinale et al. 2012; Harfoot et al. 2014). Together these deterministic environmental shifts will shape the strength and direction of any MB–EF relationships (positive, neutral, or negative) through altered patterns of taxonomic, functional, and phylogenetic diversity. Existing modeling applications provide the template for including deterministic changes into MB–EF analyses (Loreau 2010; Mokany et al. 2012). This could be achieved by integrating spatiotemporally explicit projections for future environmental conditions, macroecological diversity patterns, ecosystem functions, and management strategies.

**Figure 1:**
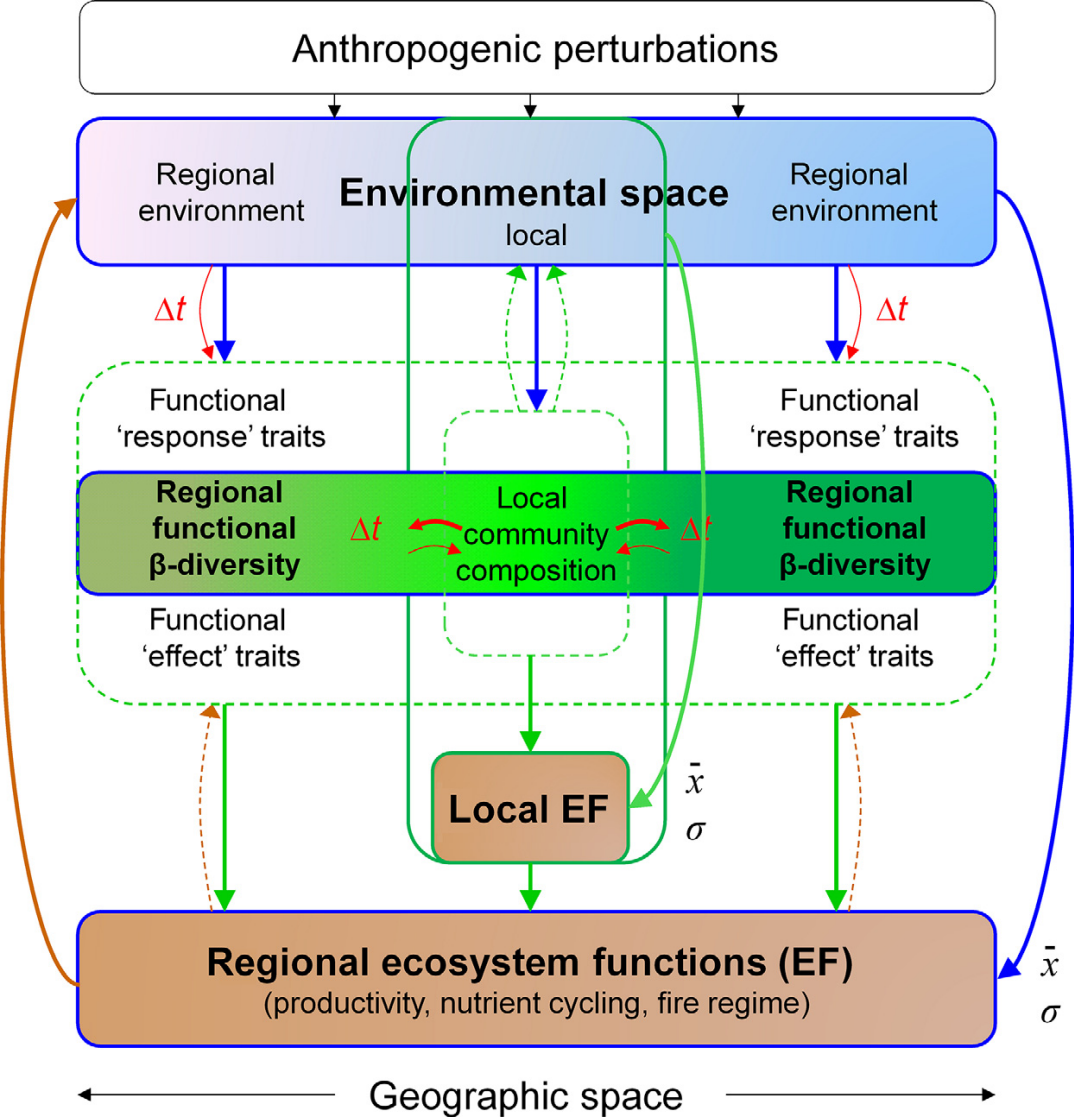
Current *β*‐diversity patterns (green box) are shaped by environmental conditions. Ecosystem functions (brown boxes and arrows, EF) are produced by biological–environmental interactions at local and regional scales. These interactions are facilitated by the distribution of particular phenotypes (i.e., functional traits) within communities, which allow species to respond to environmental conditions and influence ecosystem functions. Functional *β*‐diversity may then influence the magnitude and stability ($\overset{¯}{x}$, *σ*) of ecosystem functions at local and regional scales, under both current conditions and environmental change (Δ*t*).

Following this logic, MB–EF relationships should also be analyzed across multiple biological dimensions – taxonomic, functional, and phylogenetic – to robustly test the relevant macroecological processes and mechanisms assumed within these models. Local‐scale research suggests that mechanistic B–EF links arise primarily through the diversity and composition of functional “response” and “effect” traits (i.e., particular phenotypes) that influence how biota respond to environmental conditions and influence ecosystem functions, respectively (Mori et al. 2013). Here, it must be emphasized that trait categories are not mutually exclusive, being influenced by intraspecific variation, and are best characterized as overlapping continuums (Table 1). Nonetheless, current ecosystem functions are by their very nature facilitated by the spatial distribution of particular phenotypes. Indeed, current functional *β*‐diversity should reflect contemporary phylogenetic patterns (Wang et al. 2015) and will effectively shape functional *α*‐diversity (Fig. 1). Thus, we may expect functional *β*‐diversity to strongly influence how macroecological biodiversity responds to various directional global change scenarios across space and time (Corlett and Westcott 2013). Similarly, traits should also influence the effect of macroecological biodiversity distributions on regional‐scale ecosystem functions. However, initial MB–EF analyses have considered only compositional (i.e., taxonomic) *β*‐diversity (Pasari et al. 2013; Wang and Loreau 2014). From a macroecological perspective, we expect that the maximum heights, leaf areas, growth rates (effect traits), and fire syndromes (response traits) of dominant tree species will change across broad gradients of altitude, temperature, and precipitation. These shifts will generate patterns of functional and phylogenetic *β*‐diversity. Again, such changes could entail positive, negative, or neutral impacts on the magnitude and stability of ecosystem functions. For example, if trees with unproductive phenotypes replace each other in sections of the gradient under particular environmental change scenarios, little appreciable impact on stand biomass would be expected. Utilizing the best available information on functional and phylogenetic diversity in a spatially explicit manner across broad extents should therefore help improve the mechanistic basis for MB–EF analyses.

Modeling of MB–EF relationships in real ecosystems also needs to focus more explicitly on ecosystem functions directly relevant to planning and management decisions for global environmental change and biodiversity loss. The impact of directional bioclimatic changes on the future magnitude and stability of particular ecosystem functions will vary for different areas within biogeographic regions, and across entire continents. For example, altered streamflow regimes will affect clean water yields differently at particular points across forested catchments (Milly et al. 2005; Schelker et al. 2014). Similarly, stand‐level biomass from different forests and woodland sites across continental environmental gradients of altitude, temperature, and precipitation will not be uniformly affected under deterministic bioclimatic change scenarios (Paquette and Messier 2011; Morin et al. 2014; Ruiz‐Benito et al. 2014). At the same time, conservation strategies often focus on promoting the persistence of all native species across large regions, through targeted habitat protection, restoration, and threat minimization (Wilson et al. 2011; Pulsford et al. 2012). The most useful information for managing biodiversity and ecosystem functions under global change scenarios will therefore be spatiotemporally explicit predictions (Lindenmayer et al. 2012), encompassing fundamental MB–EF links. Several ecosystem functions can now be either measured or modeled with reasonable accuracy across broad spatiotemporal extents, such as primary productivity, evapotranspiration, and fire regime (Haverd et al. 2013; Donohue et al. 2014; Fang et al. 2015). These data provide opportunities to robustly calibrate MB–EF models with consistent measurements at relatively fine resolutions. The next section describes two key MB–EF hypotheses that offer potential to better integrate the required data inputs and outputs for testing these ideas across broad spatiotemporal extents.

## A Broader View of MB–EF Relationships in Natural Ecosystems

Building on the recent work of Wang and Loreau (2014), here we outline two broad mechanisms that may underpin MB–EF relationships in natural systems: (1) “macroecological complementarity” and (2) “macroecological spatiotemporal compensation.” These mechanisms provide testable hypotheses for how macroecological biodiversity could interact with environmental conditions to influence ecosystem functions under both current conditions and directional environmental change.

### Macroecological complementarity

**Figure 2:**
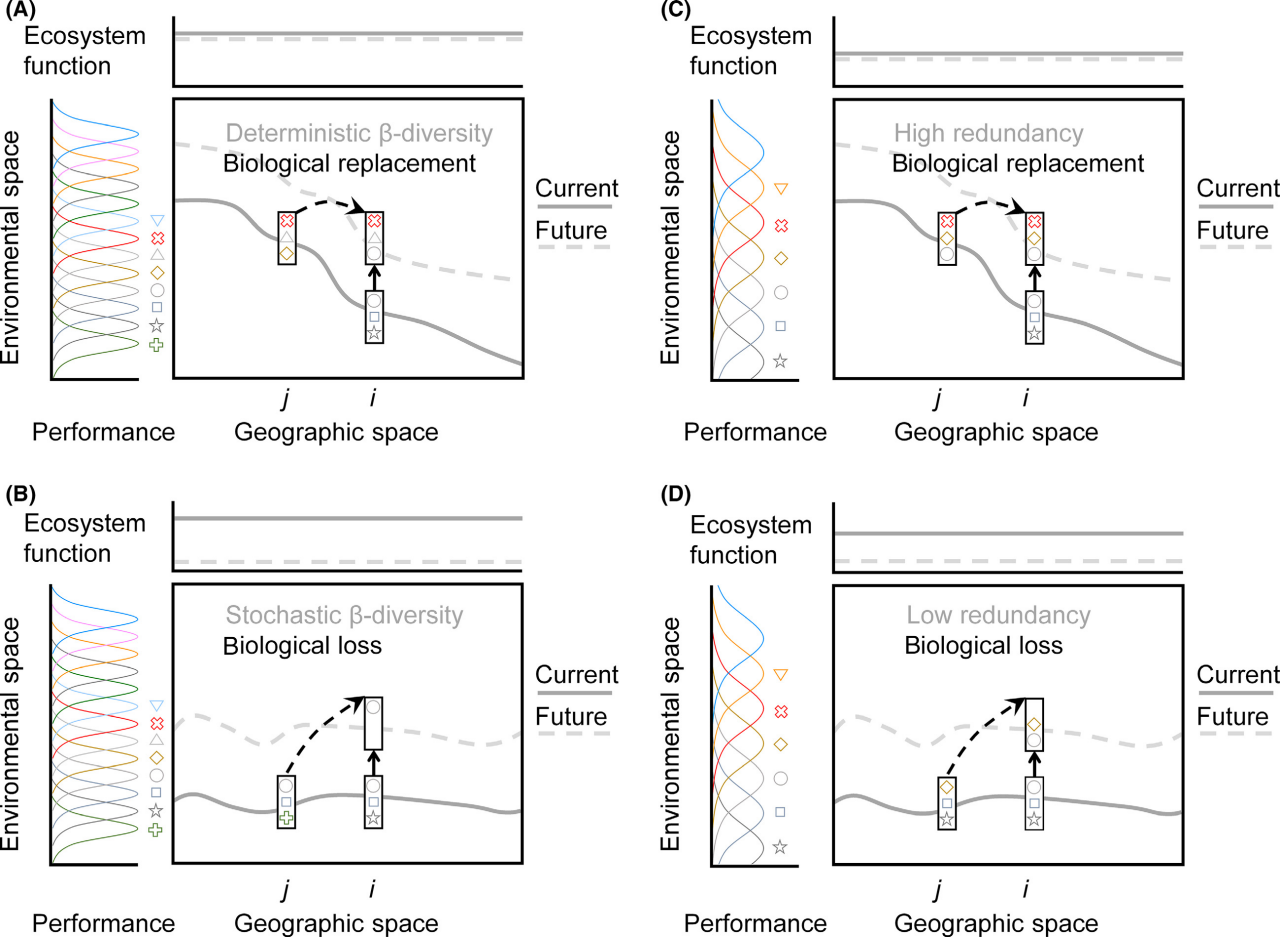
Conceptual depiction of the proposed MB–EF mechanisms. Under “macroecological complementarity,” regions with high *β*‐diversity resulting from the evolution of species with strong physiological specialization and performance in particular environments (i.e., “deterministic *β*‐diversity,” A) have high local ecosystem function (e.g., primary productivity) under current environmental conditions (current*,* dark gray lines in top ecosystem function panels). Narrow colored niches and symbols in the central panels denote species, and black rectangular boxes denote communities. Conversely, lower *β*‐diversity regions where more generalist species dominate (broader colored niches and symbols, C, D) may have relatively lower current ecosystem function (dark gray lines in top ecosystem function panels). Under “spatiotemporal compensation,” the maintenance of ecosystem function across broad scales of space and time depends on interactions between the degree and nature of phenotypic and niche specialization within the region and changing environmental conditions (future, lighter dashed gray lines in top and central plots of each panel). These interactions determine the capacity of suitably adapted species to replace less well‐adapted species under directional environmental change (biological replacement, denoted by dashed black arrows between communities *j* and *i*). Regions where *β*‐diversity has formed through physiological specialization may retain higher ecosystem function because species replacement occurs (dashed gray light lines for future in top panel, A), but could experience a greater decline in ecosystem function where biological loss occurs (“stochastic *β*‐diversity,” B).

To understand how macroecological biodiversity patterns may influence current ecosystem functions across large regions, we need to consider the different biogeographic processes responsible for shaping these patterns and their contrasting implications for ecosystem functions. Although the causes of specific macroecological biodiversity patterns remain controversial (Kraft et al. 2011), they are clearly influenced by interactions between contemporary and past environments, the dispersal abilities of organisms, and the relative strength of biogeographic barriers (Jackson and Sax 2010; Fernandez‐Going et al. 2013). Thus, it is important to consider biogeographic history when reframing macroecological diversity as an explanatory, rather than response, variable (Table 1). For example, high regional *β*‐ and *γ*‐diversity could be produced by adaptive processes such as niche specialization (Chase and Myers 2011) through strong competition within stable environments over long evolutionary timescales (Fig. 2A). Under these circumstances, we expect to observe strong relationships between species' genotypes and phenotypes, and their ability to persist and thrive in particular environments. Adaptively assembled macroecological biodiversity patterns could then influence current ecosystem functions through *environmental niche specialization* at the metacommunity scale or “macroecological complementarity.”

The “niche complementarity” hypothesis was originally framed at the local spatial scale (Table 1), predicting that greater taxonomic *α*‐diversity approximates more diverse resource‐use strategies, thereby enhancing the efficiency of ecosystem functions (Loreau 1998; Petchey 2003; Ruiz‐Benito et al. 2014). We can extend complementarity to the spatial dimension by considering the effects of *β*‐ and *γ*‐diversity. To hypothesize mechanistic relationships between ecosystem functions and macroecological biodiversity, we must assume that *β*‐diversity patterns have formed through deterministic evolutionary processes (Chase and Myers 2011). If regional *β*‐diversity has been deterministically structured, it will reflect the degree of phenotypic (i.e., physiological) optimization to current conditions through niche partitioning of environmental space (Devictor et al. 2010; Zuppinger‐Dingley et al. 2014). In such regions, collections of narrower environmental niches (higher *β*‐diversity, Fig. 2A and B) could result in more efficient performance of ecosystem functions at any point in environmental space (Baltzer et al. 2007) than regions where broader niches dominate (low *β*‐diversity, Fig. 2C and D).

High macroecological biodiversity (i.e., *β*‐ and *γ*‐diversity) can also result from more neutral processes operating within fluctuating environments over shorter evolutionary timescales (Chase and Myers 2011). Under these conditions, current environmental gradients may be fluctuating or weak, reducing the potential for niche specialization. However, events such as the formation of biogeographic barriers may generate high spatial *β*‐ and *γ*‐diversity simply through the reduced dispersal (i.e., vicariance), without necessarily leading to strong phenotypic adaptation to particular niches. In reality, the forces shaping macroecological biodiversity patterns ‐ niche specialization and vicariance ‐ are unlikely to have operated independently. Instead they have interacted across space and time, generating the different levels of current *β*‐diversity.

Because the macroecological complementarity mechanism is underpinned by phenotypic optimization, MB–EF links should arise from the patterns of functional “effect” traits that influence ecosystem functions, such as plant height and leaf size. These patterns reflect the evolutionary processes that generate phenotypic variation among organisms (Cadotte et al. 2011; Siefert et al. 2013), allowing them to exploit different niches and influence ecosystem functions. For example, the evolution of diverse plant leaf shapes affected growth and reproduction (Nicotra et al. 2011), facilitating the colonization and partitioning of new environments. The strength of relationships between functional *β*‐diversity and current ecosystem functions should then help to reveal the relative influences of adaptive and neutral processes in shaping contemporary *β*‐diversity patterns. Assuming primarily adaptive processes, we hypothesize that more biologically heterogeneous biogeographic regions, populated by physiological specialists, will display greater magnitudes of ecosystem functions under current conditions than homogenous regions where generalists dominate (Fig. 2). The *macroecological complementarity* of species' physiological functioning across metacommunities, as approximated by *β*‐ and *γ*‐diversity, represents the first mechanism through which contemporary macroecological biodiversity patterns could influence the current ecosystem functions.

### Macroecological spatiotemporal compensation

Macroecological biodiversity could also influence ecosystem functions in a more dynamic manner through the modulating effects of biological heterogeneity across space and time as environmental conditions change. The “spatial insurance” hypothesis (Loreau et al. 2003, Shanafelt et al. 2015) lays the foundation for understanding the dynamic MB–EF relationships and was recently advanced by a statistical model for “ecosystem stability in space” (Wang and Loreau 2014). This new framework partitions the stability of ecosystem functions into local, spatial, and regional components akin to the partitioning of compositional diversity (Whittaker 1960), but does not empirically quantify relationships between macroecological biodiversity and ecosystem functions. By implication, high regional *β*‐ and *γ*‐diversity is thought to facilitate spatiotemporal biological exchanges (Loreau et al. 2003) between local communities within a region when environments change, promoting regional stability of ecosystem functions (Wang and Loreau 2014). However, this prediction may only hold when dispersal is nonlimiting, given its influence on the diversity and composition of local communities over time (Matthiessen and Hillebrand 2006).

The new spatial ecosystem stability framework proposed by Wang and Loreau (2014) provides a valuable foundation for advancing MB–EF research. However, it needs to be extended in several respects to allow plausible, empirical testing across large regions. One key extension is to consider the effects of *β*‐diversity on the *magnitude* of ecosystem functions, in addition to the focus on stability adopted by Wang and Loreau (2014). Both stability and the absolute magnitude of ecosystem functions are important in natural resource management, particularly for deriving ecosystem services from particular functions (Table 1). For example, the taxonomic, functional, and phylogenetic *β*‐diversity in different forest systems across broad environmental gradients may provide the same variability of primary production under climate change simulations, but different overall magnitudes. Similarly, a broader dynamic MB–EF mechanism should explicitly consider the effects of spatiotemporal compositional differences between interacting communities on ecosystem functions. These compositional differences facilitate the dispersal between communities of new species with different physiological responses as environmental conditions change [Fig. 2A and C, Mokany et al. (2013a), Mori et al. (2013)]. Aggregated regional estimates of MB–EF relationships (Pasari et al. 2013; Wang and Loreau 2014) omit the effect of these interactions on the magnitude and stability of ecosystem functions at individual locations within regions. This is an important distinction given the inherently spatiotemporal nature of conservation planning for global change, whereby managers must allocate increasingly scarce resources between and within regions (Pressey et al. 2007; Kujala et al. 2013).

Another important extension of the spatial stability framework will be to consider the effects of directional spatiotemporal environmental change (Oliver et al. (2015), Section “Making MB–EF Relationships Applicable to Real Ecosystems”), alongside the relatively stochastic changes already considered (Wang and Loreau 2014). Directional bioclimatic shifts are already occurring and are likely to strengthen. Thus, we suggest extending the spatial stability framework to consider a broader mechanism of *macroecological spatiotemporal compensation* (Fig. 2), incorporating directional change in both environmental conditions and biodiversity distributions into predicted outcomes. Under directional change, MB–EF links could be positive, negative, or neutral depending on the ecological context, particularly the taxa, spatiotemporal extent, and resolution considered. Moreover, demonstrating macroecological complementarity is not a prerequisite for testing the spatiotemporal compensation mechanism, given the strong likelihood that significant shifts in macroecological diversity patterns will affect ecosystem functions under rapid environmental change.

Dynamic MB–EF relationships will also depend on how the biological composition of a region is impacted by environmental change, including human modifications. Functional *β*‐diversity should thus be fundamental to dynamic MB–EF links, reflecting inherent spatiotemporal trade‐offs (Mori et al. 2013; Oliver et al. 2015) in species physiological responses (e.g., fire and drought tolerance) and their effects on key ecosystem functions (e.g., gross primary productivity, evapotranspiration, fire intensity). Because gradients of human modification disproportionately impact particular environments, phenotypes, and genotypes (Laliberté et al. 2010), they will interact with climatic changes to shape future MB–EF relationships. For example, rapid, directional shifts in environmental conditions could have strong impacts on the biodiversity of noncontiguous rainforest metacommunities in the Australian Wet Tropics, due to the inhibited dispersal resulting from land clearing (Williams et al. 2009). The finely adapted functional traits and genes endemic to regions with high *β*‐diversity could fail to migrate or adapt by virtue of their specialization and isolation (Feeley and Rehm 2012). The temporal dynamics of *β*‐diversity would then be expressed through loss of local functional *α*‐diversity. High *β*‐diversity therefore also has the potential to reduce the magnitude and stability of future ecosystem functions (Fig. 2), if niche differentiation becomes disadvantageous under rapid environmental change.

## An Example: Potential MB–EF Relationships for Trees Across an Elevation Gradient

To illustrate the importance of ecological context to the proposed macroecological complementarity and spatiotemporal compensation mechanisms, we consider a simple practical example of tree communities across an altitudinal transect in southeastern Australia. We first downloaded georeferenced occurrence records from the Atlas of Living Australia (ALA, www.ala.org.au) for the 30 most common tree species known to occur along this transect in southeastern Australia (see Appendix S1, Table S1). Our coastal–inland transect was 1 km × 500 km, centered on latitude −36.48 to encompass a broad range in elevation, temperature, and precipitation. This transect crosses six of Australia's 85 continental Interim Biogeographic Regions [IBRAs, see http://www.environment.gov.au/land/nrs/science/ibra, (Thackway and Cresswell 1997)] and 18 of Australia's 23 major vegetation groups (see www.environment.gov.au/land/native-vegetation/national-vegetation-information-system, Figure S1). This study system is primarily comprised of Eucalypt‐dominated forests and woodlands – specifically *Eucalypt Open Forest* and *Woodland* vegetation groups – where taxonomic diversity can be calculated with reasonable accuracy in the Australian context. Species records were restricted to those observations with spatial errors <2 km, occurring within native vegetation and recorded since 1970 (e.g., biocache.ala.org.au/occurrences/search?q=Angophora%20floribunda&fq).

**Figure 3:**
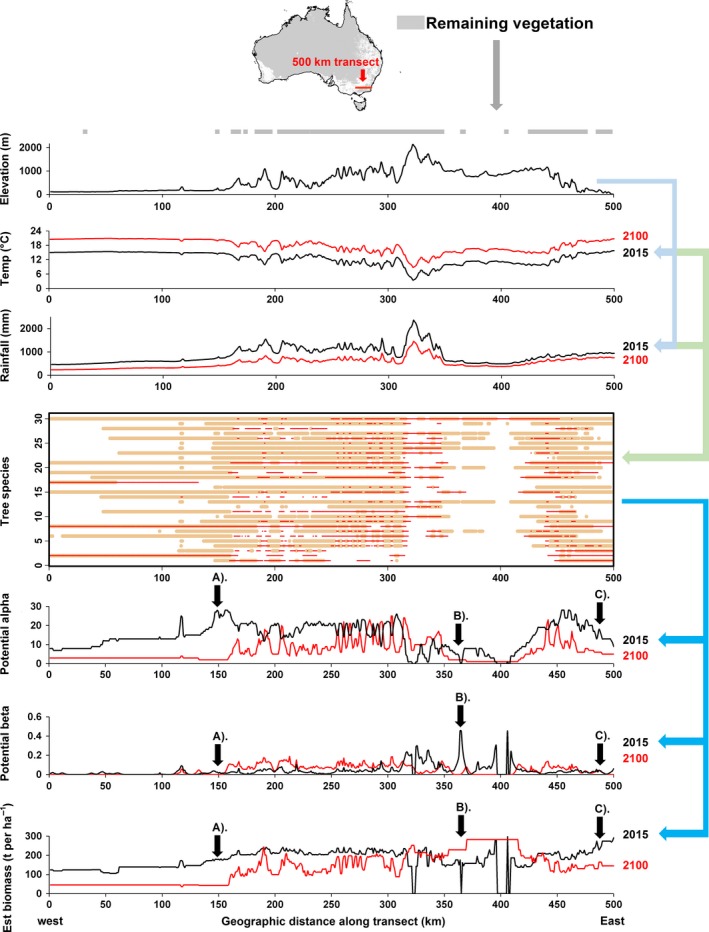
A simple case study illustrating the importance of ecological context to the macroecological complementarity and spatiotemporal compensation mechanisms, using 30 key tree species and tree stand biomass (tonnes per ha^−1^, bottom panel). The top map in gray shows the 1‐km extant vegetation mask for all of Australia. The gray strip depicts the remaining vegetation across our case study system: a 500 km × 1 km altitudinal transect in southeastern Australia (red line on map). Current (2015, black lines) and future (2100, red lines) temperature (°C) and precipitation (mm) are plotted at each point along this transect. Variations in elevation, temperature, and precipitation from east to west drive changes in current environmental conditions, and subsequent variations in species distributions (light blue, green, and darker blue arrows connecting environmental conditions, species distributions, potential biodiversity values, and estimated biomass). The beige lines in the central panel (“tree species”) represent the current (2015) predicted occurrences of each tree species in each transect cell according to their convex hulls, and the red lines represent the predicted future occurrences (2100). Current (2015, black lines) and future (2100, red lines) potential species richness (potential alpha) and potential species turnover (potential beta) are also plotted at each transect point, estimated from the occurrence records for all 30 tree species.

The spatial species records were then used to fit simple convex hulls for each species as a function of the current (1990) mean annual temperature (°C) and precipitation (mm) at the record locations. These values were derived from 1‐km resolution interpolated climate surfaces for the Australian continent (see www.emast.org.au). Convex hulls were fitted with the alphahull R package, version 1.0 [R version 3.1.2, (R Core Team 2014)]. This method was used because the analysis outcome was the presence or absence of tree species in each transect cell. Convex hulls were thus useful for making simple predictions of the current and future distribution of each species along the 500‐km transect using the presence‐only ALA data (e.g., Figure S2, Appendix S1). Dispersal limitations were incorporated into future distribution predictions by limiting the 2100 distributions to within 5 km (i.e., 5 grid cells) of the current occurrence records. The current (2015) and future occurrences (2100) of each tree species in each cell are plotted in the central panel of Figure 3 in beige and red, respectively (see “tree species” panel, Fig. 3).

The predicted occurrences of all 30 tree species in each grid cell of the 500‐km transect were then combined to create *α*‐diversity estimates for each cell by summing the species occurrences columns for each row. Community aboveground biomass estimates (t ha^−1^) were predicted by averaging the mean stand biomass for all tree species either *potentially* present (2015) or predicted to occur (2100), in each transect cell. See appendix S1, Table S1 for biomass estimates for each species (Grierson et al. 1992; Turner et al. 1999; Keith et al. 2000; Raison et al. 2003). Biomass estimates will therefore be affected by changes in the stand structure and successional status of the particular Eucalypt forest or woodland that each cell occurs in. We then used the predicted species composition for each transect cell to create a site × site matrix of Sørensen dissimilarity values (Sørensen 1948) for pairs of sites along the transect using the betapart R package [version 1.3, (Baselga 2010), R version 3.1.2, (R Core Team 2014)]. These pairwise dissimilarity values were used to calculate the average pairwise *β*‐diversity within a five‐km radius surrounding each transect cell. This distance was chosen to match the maximum dispersal distance specified in the species distribution shifts.

**Figure 4:**
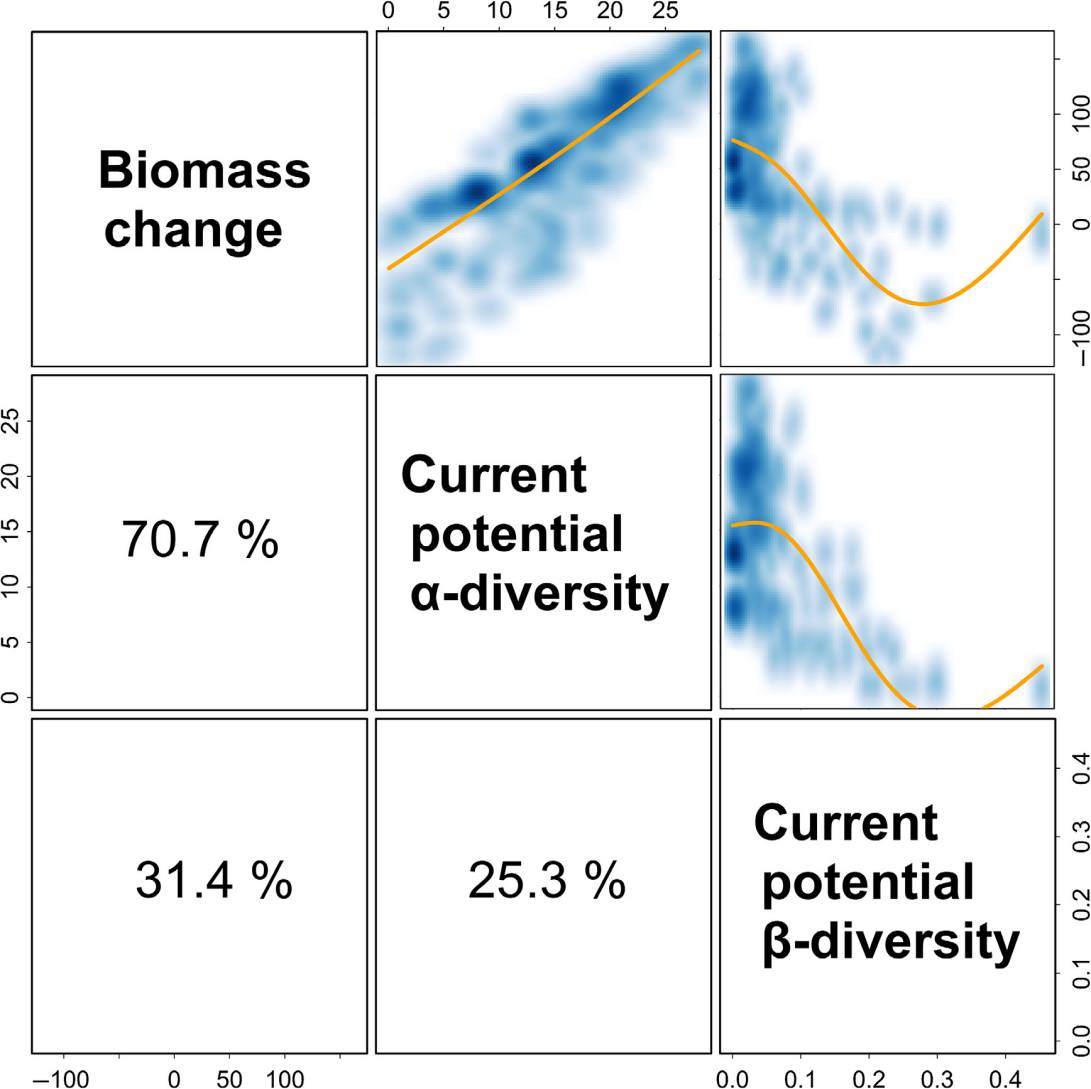
Plot of stand biomass change for all 500 cells in the altitudinal transect (t ha^−1^ as estimated from the species occurrences) against current potential *α*‐ and *β*‐diversity. *α*‐diversity values are counts of species, and *β*‐diversity values are the Sørensen dissimilarity (between 0 and 1). Deviance explained values (%) for generalized additive models of each plot using four knots are displayed in the left half of the panel (orange lines are the fitted spline regressions).

Changes in altitude and increasing aridity from east to west across the 500‐km transect drive spatial turnover in current environmental conditions (indicated by the light blue, green and darker blue arrows connecting the top three panels in Fig. 3). Current environmental turnover then generates changes in the occurrences of tree species between transect cells, determining the potential current *α*‐diversity of each cell and potential *β*‐diversity surrounding each cell (Fig. 3, potential alpha and potential beta). The estimated stand biomass of each cell is thus a by‐product of these macroecological patterns. At transect point A, high potential *α*‐diversity and low potential *β*‐diversity are calculated from the species occurrences under current environmental conditions, associated with a substantial decrease in future biomass when rainfall decreases and temperature increases. In contrast, at point B on the edge of the Great Dividing Range, the species occurrences generate low potential *α*‐ and high potential *β*‐diversity, maintaining future biomass (Fig. 4, current potential *β*‐diversity vs. biomass change). Closer to Australia's east coast at point C, high current potential *α*‐diversity and low potential *β*‐diversity are also associated with a sharp decrease in future biomass. Although there are two clear peaks in *β*‐diversity surrounding each cell (e.g., Fig. 3, transect point B), few or no species are estimated to occur there under current conditions. The predicted species occurrence records (middle panel of Fig. 3) show that spatial turnover in this part of the gradient is between small numbers of moderately productive Eucalypts (e.g., *E. Elata* and *E. Vimalis* shift to point B for 2100). Although the decreases are larger for *α*‐ than *β*‐diversity under future conditions, in large parts of the altitudinal gradient both diversity measures increase (Fig. 3, 200–300 km).

Our example highlights that current macroecological patterns of species distributions influence current ecosystem functioning, while also shaping future biodiversity patterns – hence affecting future ecosystem functioning. However, the direction and nature of these relationships will vary across the gradient, depending on the ecological context. Steep directional environmental change (i.e., increasing temperature and decreasing rainfall), the particular plant phenotypes and genotypes occurring in each cell, and land‐use patterns all interact to determine stand biomass change in our simple demonstration (Fig. 4). Such variations of ecological context should therefore be integrated into predictions of the consequences of macroecological biodiversity patterns for current and future ecosystem functions. For example, the dominant tree species across the gradient vary considerably in their ability to sequester carbon (see Appendix S1, Table S1). If the biological turnover in sections of the gradient is between the small numbers of trees with unproductive phenotypes (e.g., slow growth rates, low maximum heights, and smaller leaf areas), plausible MB–EF models should not predict significant biomass change. Previous analyses have attempted to encapsulate ecological context by either simulating the hypothesized relationships, or using fine‐scale experiments with little application to natural ecosystems. However, the argument that preserving different spatial and temporal dimensions of diversity is necessary to maintain ecosystem function under environmental change can only be supported using empirical analyses across broad spatiotemporal scales.

## Potential Avenues for Testing MB–EF Mechanisms in Real Ecosystems

To thoroughly account for ecological context in MB–EF mechanisms across broad scales, empirical analyses must overcome a long‐standing challenge in ecology: the effect of spatiotemporal nonstationarity on ecological data. Nonstationarity is not a new concept in geography (Tobler 1970; Fortin and Dale 2005; O'Sullivan and Unwin 2010), but needs greater consideration in ecosystem function research. The inherent context dependence of MB–EF links – arising from biogeographic history and directional environmental change – means that relationships will vary spatially, temporally, and with scale, forming complex emergent patterns (e.g., Figs. 3, 4). For example geographically structured environmental variation should be the primary influence on ecosystem functions relating to the carbon cycle (Kanniah et al. 2013). However, the strength of environmental influences on biological variables will vary strongly across space and time (Brunsdon et al. 1998; Miller et al. 2007; Osborne et al. 2007). Because environmental and biological influences on ecosystem functions are inter‐related, spatiotemporal nonstationarity will affect the magnitude, accuracy, meaning, and interpretation of MB–EF predictions, at both individual locations and across biogeographic regions. Although analyzing nonstationary macroecological relationships will present significant challenges, these problems are not insurmountable and can be partially addressed with existing methods and data.

There are three main avenues for plausibly testing MB–EF mechanisms in natural ecosystems. Firstly, macroecological complementarity could be tested in *geographic space* by quantifying the unique contributions of *α*‐, *β*‐, and *γ*‐diversity to ecosystem functions over and above environmental conditions at continental scales. This could be achieved by combining either empirical biodiversity data or models of *α*‐, *β*‐, and *γ*‐diversity predicted by environmental turnover across continental scales (Ferrier et al. 2007), with fine‐resolution surfaces for environmental conditions and ecosystem functions (e.g., remotely sensed gross primary productivity, Table 2). Secondly, macroecological complementarity could be tested in *environmental space* at the regional scale. This could be achieved by quantifying bivariate relationships between species and community‐level niche widths in key environmental dimensions and functional traits approximating physiological performance (e.g., plant height, leaf area, seed size, Table 2). Quantifying environmental niche widths at multiple biological dimensions (i.e., phenotypic, genotypic) could better approximate the key mechanism of ecological specialization (Devictor et al. 2010) hypothesized to underpin MB–EF mechanisms. If niche widths are strongly related to ecological performance, multivariate relationships between ecosystem functions, environmental conditions, and community‐level niche widths could then be quantified using techniques such as causal networks and spatiotemporally explicit statistical modeling (Grace et al. 2014; Lamb et al. 2014; Fotheringham et al. 2015).

**Table 2:** The main avenues, potential methods, and examples of Australian data sources for testing both MB–EF mechanisms in real ecosystems. EF denotes data sets quantifying ecosystem functions, ENV denotes environmental data sets, and BIO denotes biodiversity data sets

Avenue	Methods	Examples of Australian data sources and spatial extents
Test *macroecological complementarity* in geographic space	Apply spatially interpolated models of α‐, β‐, and γ‐diversity (Ferrier et al. 2007) to test their unique contributions to EF, over and above the contribution of environmental conditions, at continental scales.	EF: monthly continental remotely sensed gross primary productivity layers at 250‐m resolution [GPP, (Donohue et al. 2014)]. Annual potential evapotranspiration at 1‐km resolution ( www.emast.org.au). ENV: monthly continental climate surfaces at 1‐km resolution. BIO: vascular plant occurrence records at 1‐km resolution across a continent ( www.ala.org.au).
Test *macroecological complementarity* in environmental space	Quantify relationships between environmental niche widths (ENW) for individual species and ecological performance, for example, niche width along soil moisture gradients vs. plant growth. Quantify multivariate relationships between EF, environment, and community‐level ENW (cENW) using causal networks and structural equation modeling [SEM, Lamb et al. (2014)].	Proxies of physiological performance (e.g., functional traits) EF: GPP layers downscaled to 250 m. ENV: monthly continental climate surfaces downscaled to 250 m. Soil attribute layers at 90‐m resolution (Viscarra Rossel et al. 2014). BIO: vascular plant occurrence records and community survey plots (Mokany et al. 2014). Trait and phylogenetic databases (Kattge et al. 2011).
Test *spatiotemporal compensation* in geographic and environmental space under rapid environmental change scenarios	Develop and apply spatiotemporal models integrating biodiversity composition and ecosystem function (cENW, EF, and taxonomic, functional and phylogenetic α‐, β‐, and γ‐diversity). Continue developing MB–EF simulations, parameterized using macroecological data sets. Quantify multivariate relationships between EF, environment, cENW, functional traits, and phylogeny under various environmental change scenarios.	Same data as mentioned above, but must consider how to project complex relationships under environmental change scenarios (e.g., combining climate surfaces for 2100 with new biodiversity models and simulations). Long‐term ecological monitoring sites (e.g. www.supersites.net.au/supersites/fnqr).

Third, the capacity for spatiotemporal compensation and its impact on ecosystem functions under rapid environmental change could be assessed by developing and applying new simulation and modeling approaches. These methods could be built on existing applications (Loreau 2010; Mokany et al. 2012), combining mechanistic projections for both biodiversity and ecosystem functions at regional to continental scales (Table 2). Such information could potentially add capability to existing management tools for resource allocation (Wilson et al. 2011). Under rapid environmental change, adherence to a single management strategy (e.g., maximizing *α*‐, *β*‐, and *γ*‐diversity, or connectivity) is unlikely to deliver optimal outcomes in all locations (Mokany et al. 2013b). A number of regions around the world have been sufficiently well studied over space and time to robustly test the MB–EF relationships at appropriate spatiotemporal resolutions, including plant community data sets in Australia (Metcalfe and Ford 2008; Mokany et al. 2014), North America (Potter and Woodall 2014), and Europe (Schaminée et al. 2009). Combining spatially extensive vegetation survey networks such as these with functional trait databases (Kattge et al. 2011) and phylogenies provides an ideal test bed for MB–EF hypotheses. This not only includes investigating contemporary MB–EF links, but also assessing the spatiotemporal compensation mechanism in both geographic and environmental space (Table 2). Explicitly spatiotemporal, neighborhood‐based analyses that do not assume stationarity (Sengupta and Cressie 2013; Mellin et al. 2014) would thus be important to accurately quantify local variations in MB–EF relationships.

## Conclusions

The effect of macroecological biodiversity on ecosystem functions could be positive, negative, or neutral, depending on ecological context. Thus, the argument that biodiversity must be preserved at multiple spatiotemporal dimensions in order to maintain ecosystem functions under environmental change can only be tested comprehensively across broad scales. We have outlined the key conceptual and technical challenges requiring further investigation for conducting plausible, empirical tests of relationships between macroecological scale biodiversity and ecosystem functions. The likelihood that significant shifts in macroecological patterns of biodiversity will affect future ecosystem functions highlights the importance of investigating the underlying mechanisms with the most appropriate methods and data. Then, we will be better able to assess the potential role of biological diversity in maintaining broad‐scale ecosystem functions during rapid environmental change, with possible implications for more effective management strategies.

## Conflict of Interest

None declared.

## Supporting information


**Figure S1.** Maps showing (A): the 1‐km extant vegetation mask for all of Australia in grey, (B): the Interim Biogeographic regions, and (C): the Major Vegetation Groups intersected by the 1 km × 500 km transect, centered on latitude −36.48 (red line in all three maps). Scale bar applies only to panels (B) and (C).

**Figure S2.** Example plot of the convex hull fitted to the occurrence records for *Eucalyptus sieberi*, one of the 30 most common tree species in southeastern Australia used to create Fig. 3 in the main text. The x axis is annual precipitation (mm) divided by 100, so as to scale the values relative to the y axis for mean annual temperature (°C). Convex hulls were fit to all 30 species using the same methods.

**Figure S3.** Plot of current, future, and biomass change (all in tonnes per ha^−1^) against current and future *α*‐ and *β*‐diversity. *α*‐diversity values are counts of species, and *β*‐diversity values are the Sørensen dissimilarity (between 0 and 1).

**Appendix S1.** Additional table and figures for case study of MB–EF relationships for trees across an elevation gradient.
**Table S1.** The 30 most common tree species in southeastern Australia used to create Fig. 3 in the main text, showing the number of georeferenced records from the Atlas of living Australia (ALA) with a spatial error <2 km, occurring within native vegetation and recorded since 1970.
