# Supplementary figures and images for: Macroecological scale effects of biodiversity on ecosystem functions under environmental change
Source: Ecol Evol. 2016 Mar 16;6(8):2579–93. doi: 10.1002/ece3.2036 (PMC4798165; doi:10.1002/ece3.2036)

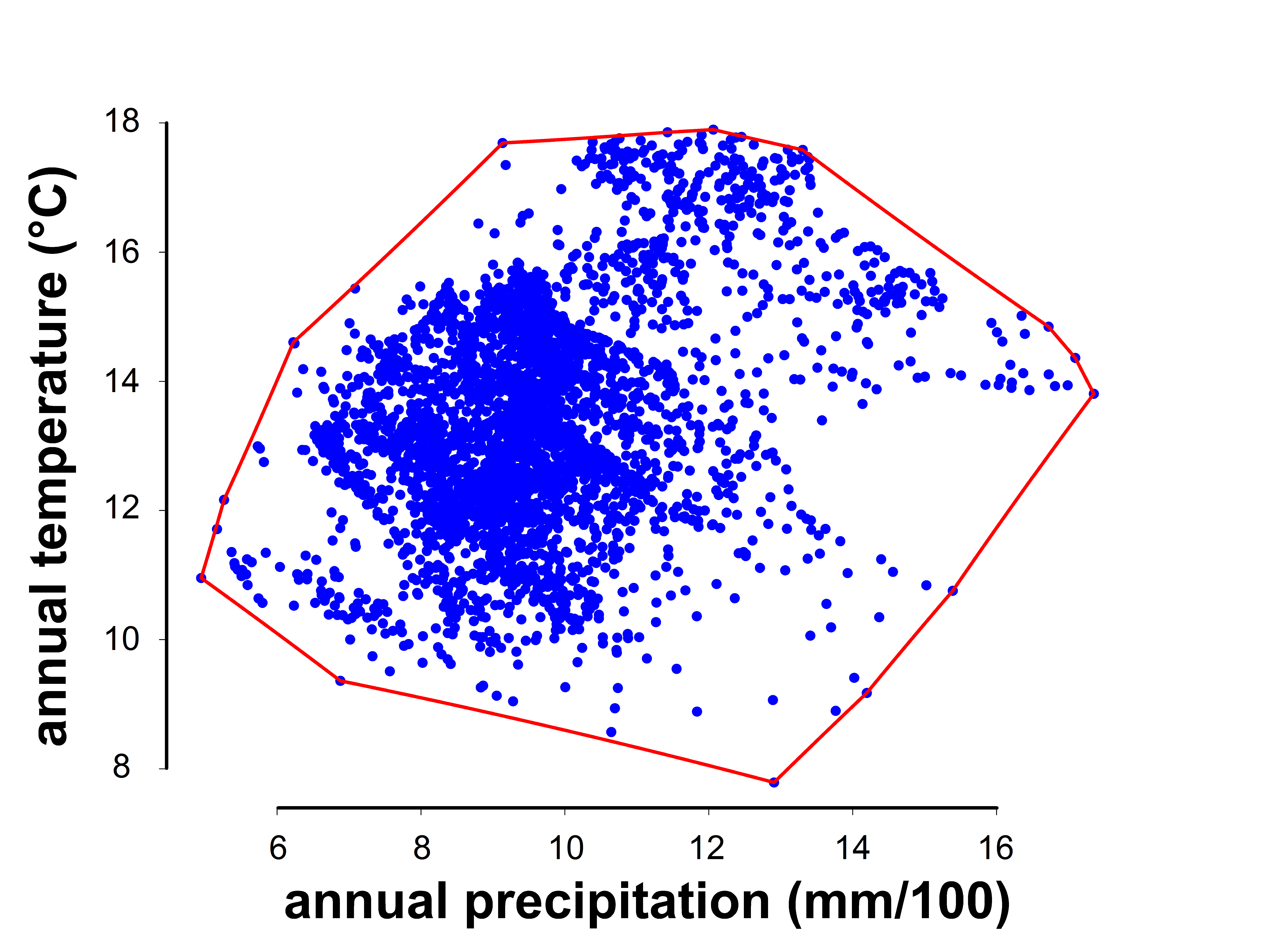

Supplement: Supplementary file 2 — Figure S2. Example plot of the convex hull fitted to the occurrence records for Eucalyptus sieberi, one of the 30 most common tree species in southeastern Australia used to create Fig. 3 in the main text. The x axis is annual precipitation (mm) divided by 100, so as to scale the values relative to the y axis for mean annual temperature (°C). Convex hulls were fit to all 30 species using the same methods. [file ECE3-6-2579-s002.png]

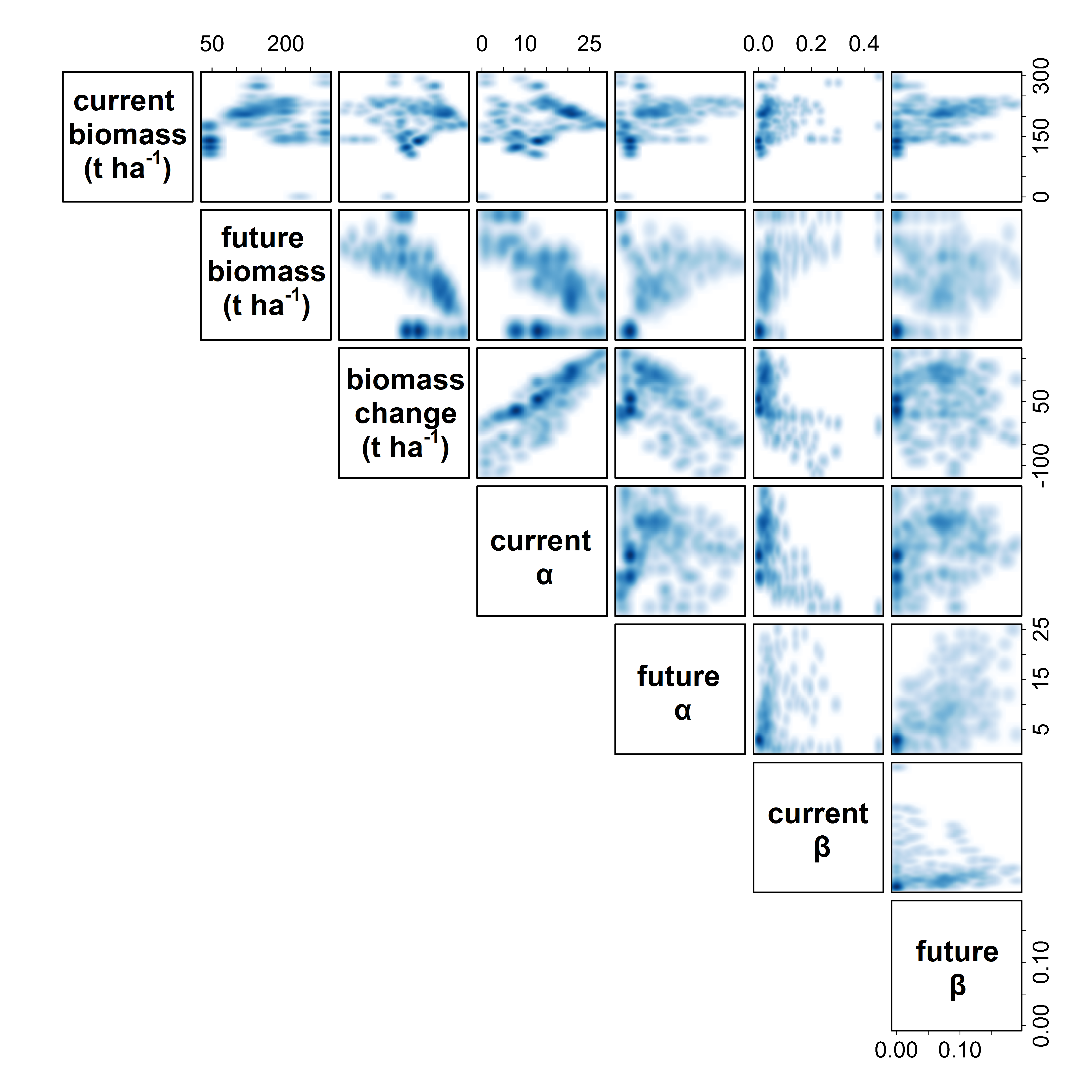

Supplement: Supplementary file 3 — Figure S3. Plot of current, future, and biomass change (all in tonnes per ha−1) against current and future α‐ and β‐diversity. α‐diversity values are counts of species, and β‐diversity values are the Sørensen dissimilarity (between 0 and 1). [file ECE3-6-2579-s003.png]
